# The antiviral protein Viperin suppresses T7 promoter dependent RNA synthesis–possible implications for its antiviral activity

**DOI:** 10.1038/s41598-018-26516-z

**Published:** 2018-05-25

**Authors:** Anna Dukhovny, Amir Shlomai, Ella H. Sklan

**Affiliations:** 1 Department of Clinical Microbiology and Immunology, Sackler School of Medicine, Tel Aviv University, Tel Aviv, 69978 Israel; 2 Department of Medicine D and the Liver Institute, Rabin Medical Center, Beilinson Hospital, Petach-Tikva, Israel; 3 Sackler Faculty of Medicine, Tel Aviv University, Tel Aviv, Israel

## Abstract

Viperin is a multifunctional interferon-inducible broad-spectrum antiviral protein. Viperin belongs to the S-Adenosylmethionine (SAM) superfamily of enzymes known to catalyze a wide variety of radical-mediated reactions. However, the exact mechanism by which viperin exerts its functions is still unclear. Interestingly, for many RNA viruses viperin was shown to inhibit viral RNA accumulation by interacting with different viral non-structural proteins. Here, we show that viperin inhibits RNA synthesis by bacteriophage T7 polymerase in mammalian cells. This inhibition is specific and occurs at the RNA level. Viperin expression significantly reduced T7-mediated cytoplasmic RNA levels. The data showing that viperin inhibits the bacteriophage T7 polymerase supports the conservation of viperin’s antiviral activity between species. These results highlight the possibility that viperin might utilize a broader mechanism of inhibition. Accordingly, our results suggest a novel mechanism involving polymerase inhibition and provides a tractable system for future mechanistic studies of viperin.

## Introduction

The interferon (IFN) response is one of the first lines of host innate defense against viral infections. Pattern recognition sensors in the infected host cell recognize the incoming pathogens and initiate signal transduction cascades that culminate in the host cell’s nucleus. These signaling cascades activate type I IFN production and the induction of interferon-stimulated genes (ISGs) aimed to prevent infection in an autocrine and paracrine manner^1^. Viperin is an IFN stimulated antiviral protein that inhibits a broad spectrum of both DNA and RNA viruses^2,3^. Interestingly, viperin was found to inhibit different viruses at different steps of the viral life cycle suggesting that this protein has multiple functions. Viperin was found to protect the infected cells by affecting lipid raft formation and modulating cellular metabolism^4,5^.

Viperin is a member of the “Radical SAM” family of enzymes^6,7^. These enzymes contain a consensus Cx3Cx2C motif responsible for binding iron-sulfur clusters to preform reductive cleavage of S-Adenosylmethionine (SAM) to generate a deoxyadenosyl radical and methionine^8^. The obtained radical is highly reactive and is known to mediate a variety of reactions^9^. No specific enzyme activity, however, has been yet assigned to viperin. The Fe-S binding motif, however, seems to be essential for its antiviral activity against some viruses^10,11^. Viperin also requires an additional protein, cytosolic iron-sulfur assembly component 1(CIAO1) crucial for the Fe/S cluster insertion and thus for its SAM activity and accordingly for its antiviral activity against the aforementioned viruses^11,12^. The broad range of viperin-affected viruses across taxonomic groups impedes the efforts to identify its specific antiviral effects. Interestingly, in many RNA viruses viperin was shown to inhibit viral RNA accumulation^11,13–18^. Moreover, viperin inhibited Hepatitis C, Dengue and West Nile virus replication from subgenomic replicons^14,17–19^. Subgenomic replicons contain only the viral non-structural proteins essential for viral replication and lack the viral structural proteins, further confirming that viperin inhibits viral replication. Mechanistically, viperin was shown to interact with non-structural proteins of hepatitis C virus and dengue virus that are essential for viral replication. These interactions are thought to be associated with its antiviral activity^13,14,17^. Here we describe a substantial inhibitory effect of viperin expression on bacteriophage T7 polymerase mediated transcription in mammalian cells. The fact that viperin inhibits transcription by a bacteriophage polymerase supports the conservation of viperin’s antiviral activity between species. Inhibition of the viral transcription might be a compelling possible mechanism for viperin’s mode of action. Several recent findings support such a hypothesis; Oxetanocin A, an antiviral nucleoside analogue produced in bacteria, was found to be biosynthesized by a radical SAM enzyme^20^. This suggests that SAM radical family enzymes can generate antiviral products. Furthermore, structural similarity studies of the active site of viperin, based on the crystal structure of murine viperin, suggest that the substrate may be a nucleoside triphosphate^21^.

Bacteriophage T7 DNA-dependent RNA polymerase (T7 polymerase) is a well-characterized RNA polymerase. T7 polymerase is widely used as a model to study transcription and in a wide verity of molecular biology applications due to its robust activity and promoter specificity^22^. T7 RNA polymerase functions in the cytoplasm and thus is the most widely used RNA polymerase in viral reverse genetic systems driving the initial transcription of RNA viruses replicating in this compartment^23,24^. Here we describe a substantial effect of viperin on bacteriophage T7 polymerase activity in mammalian cells. Overexpression of viperin strongly inhibited green fluorescent protein (GFP) expression from a T7 promoter (T7-GFP). Viperin did not inhibit RNA polymerase II dependent RNA synthesis from a Cytomegalovirus (CMV) promoter under similar conditions. This inhibition was specific and not at the level of translation. Mutagenesis of viperin’s functional domains inhibited this activity. Following T7-GFP transfection, 5′-bromouridine 5′-triphosphate (BrUTP) incorporation experiments showed significantly lower levels of RNA synthesis in the cytoplasm of viperin-expressing cells. Previous studies show that viperins are highly conserved and that fungi, bacteria, and archaebacteria express viperin-like enzymes^21^, indicating that viperin’s effect on T7 polymerase might be conserved. Taken together our results support the conservation of viperin’s antiviral activity between species and suggest a common mechanism of inhibition might be responsible of these activities.

## Results

### Viperin inhibits expression from T7 polymerase promoter

**Figure 1:**
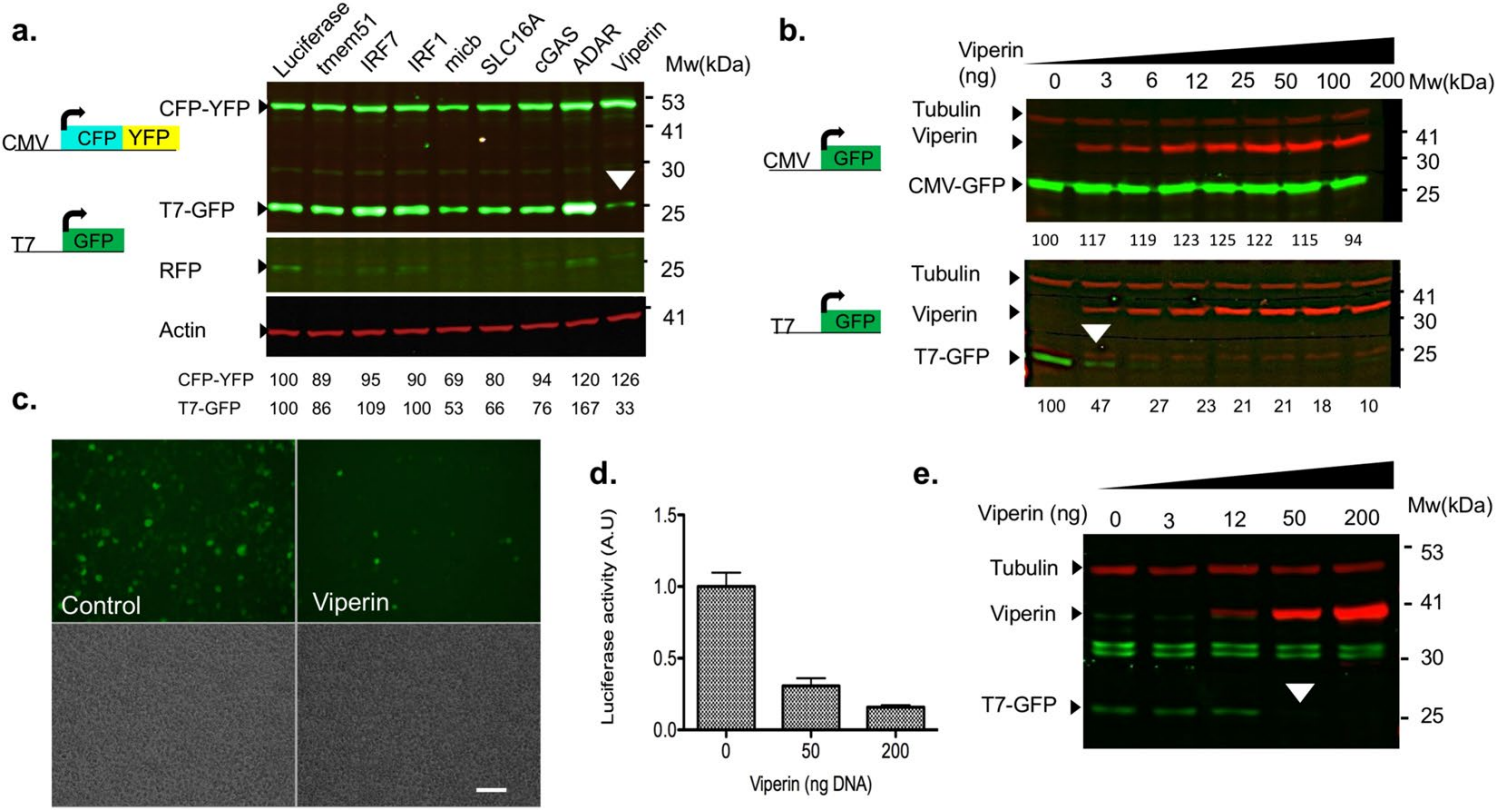
Viperin inhibits T7-GFP expression. (**a**) Viperin is the only ISG specifically inhibiting expression from T7-GFP. HEK293-T7 cells were infected with ISG expressing lentiviruses. After 24 h the cells were co-transfected with T7-GFP and a CFP-YFP fusion protein expressed from a CMV promoter. The cells were analyzed 48 h post-transfection by Western blot with a GFP antibody. RFP expression was used to confirm lentivirus infection. Actin was used as a loading control. Bands showing the effect of viperin on T7-GFP are labeled with white arrow head. Quantification of the bands compared to luciferase control and normalized to actin is shown at the top. (**b**) Viperin inhibits T7-GFP expression in a dose dependent manner. HEK293-T7 cells were transfected with the indicated amounts of viperin-expressing plasmid. After 24 h the cells were transfected with a T7-GFP or CMV-GFP. The cells were lysed after 48 h and analyzed by Western blot using GFP, tubulin and viperin antibodies. Quantification was performed as described above and is shown at the bottom of each panel. Presented is a representative gel from at least 3 independent repeats. (**c**) Viperin inhibits T7-GFP expression. HEK293-T7 cells were transfected with viperin (50 ng/well). The next day the cells were transfected with T7-GFP. Images were obtained 48 h after transfection. Bar = 100 µM. Presented are representative images from at least 5 independent repeats. (**d**) Viperin inhibits T7-Luciferase expression. HEK293-T7 cells were transfected with the indicated amounts of viperin. The next day the cells were co-transfected with T7-Firefly luciferase (0.8 µg/well) and ß-galactosidase (10 ng/well) expressing plasmids. The cells were lysed 48 h post-transfection and analyzed for luciferase and ß-galactosidase activity. Presented is representative graph from at least 5 independent repeats preformed in triplicates. (**e**) Viperin inhibits T7-GFP expression from ectopically expressed T7 polymerase. HEK293T cells were co-transfected with increasing amounts of viperin together with T7-GFP and T7 polymerase. The cells were lysed and analyzed by Western blot with GFP (green) and viperin (red) specific antibodies 24 h post-transfection. Tubulin (red, top) was used as a loading control. Full-length blots are presented in Supplementary Fig. 1.

While conducting a screen for host factors affecting a virus using a T7-polymerase based reverse genetics system, viperin was identified as a potent T7 polymerase inhibitor. In this screen, we expressed various ISGs from a bicistronic lentivirus also expressing Red fluorescent protein (RFP) as an infection control^25^. A plasmid containing Renilla luciferase was used as a control. Overexpression of viperin strongly inhibited the expression of T7-GFP. Interestingly, this inhibition was specific for expression from the T7 promoter and did not affect the expression of a cyan fluorescent protein (CFP)- yellow fluorescent protein (YFP) fusion from an RNA polymerase II-dependent CMV promoter (Fig. 1a). Of note, micb affected T7-GFP expression levels to some extent. However, this reduction was also observed on CFP-YFP expression indicating a less specific effect. Thus, this inhibitory effect was specific to viperin and did not occur upon expression of any other ISG (Fig. 1a). A different ISG, adenosine deaminase acting on RNA (ADAR1), elevated the expression of T7-GFP (Fig. 1a). This is consistent with ADAR1 role in increasing gene expression at the translational level, by decreasing protein kinase PKR-dependent eIF2α phosphorylation^26^. To further confirm this result, we transfected HEK293 cells stably expressing T7 polymerase (HEK293-T7) with increasing levels of viperin and tested its effect on GFP expression from a T7 or CMV promoters (Fig. 1b). A dose dependent inhibition of T7-GFP, but not of CMV-GFP, was observed in transfected cells (Fig. 1b, bottom panel). Images from HEK293-T7 cells co-transfected with viperin followed by transfection with T7-GFP show strongly reduced GFP fluorescence in the viperin-transfected cells as compared to mock-transfected control (Fig. 1c). To confirm that this is neither a GFP-specific nor a GFP-expression vector related phenomenon, we repeated the experiment using a luciferase-based assay utilizing firefly luciferase under the control of a T7-promoter and a ß-galactosidase expression plasmid as a transfection control (Fig. 1d). Viperin strongly inhibited the luciferase activity in a dose dependent manner. These results establish that viperin strongly and specifically inhibits expression from a T7 polymerase promoter. To verify that the obtained effect is not cell line dependent we co-transfected T7 polymerase together with T7-GFP and increasing amounts of viperin into HEK293T cells. Viperin strongly inhibited T7-GFP in a dose dependent manner (Fig. 1e). Similar results were obtained in BSR cells (a baby Hamster Kidney (BHK)-21 clone stably expressing the T7 polymerase^27^, not shown).

### Specific mutations in viperin abolish its inhibitory effect on T7-GFP expression

**Figure 2:**
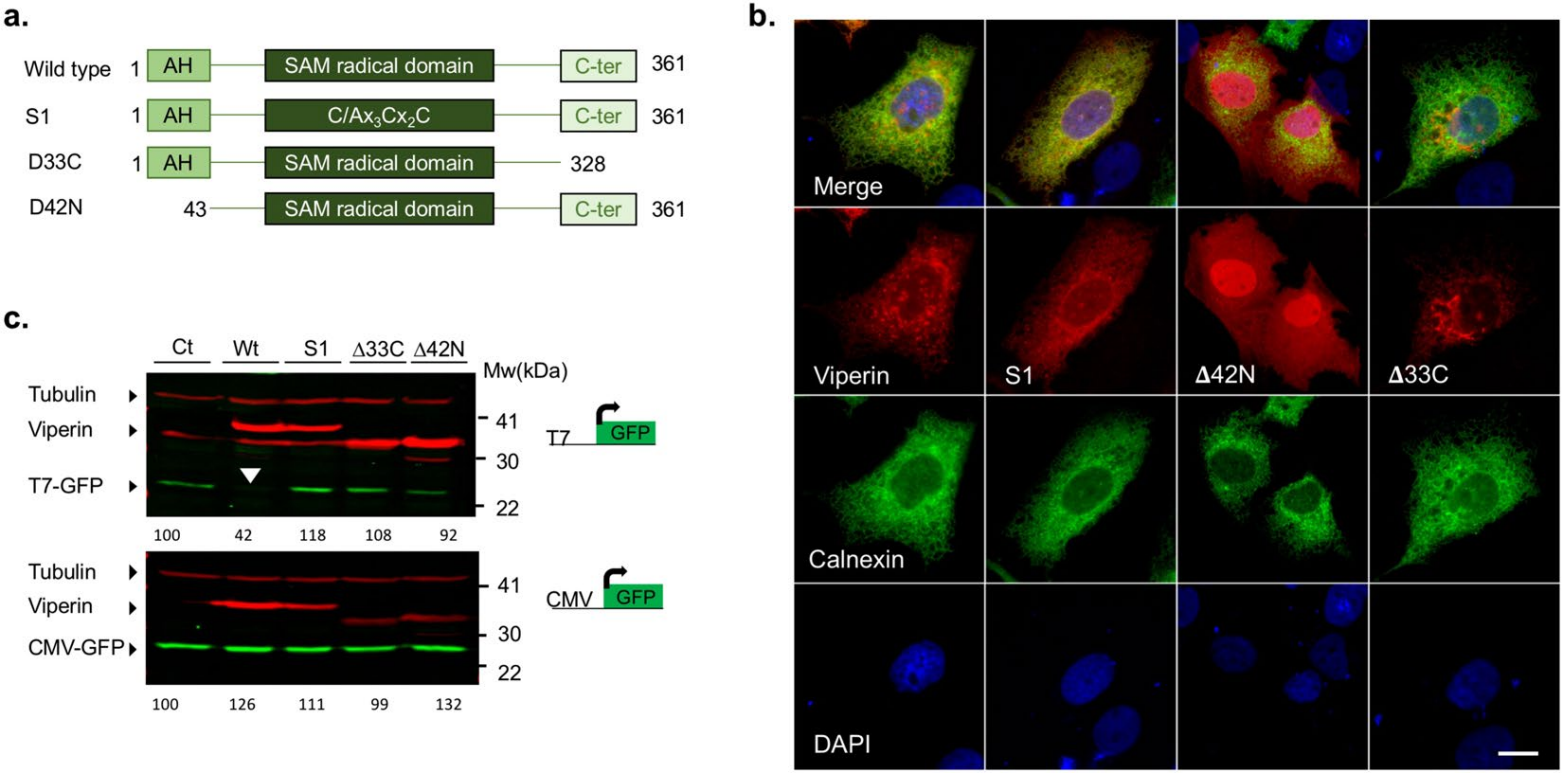
Mutations in the functional domains of viperin abolish its effect on T7-GFP expression. (**a**) Schematic representation of the viperin mutants used. (**b**) Subcellular Localization of the viperin mutants. Viperin mutants were co-transfected into Huh7 cells with a plasmid expressing calnexin-GFP as an ER marker. The cells were fixed 24 h post transfection and immunostained using a viperin antibody. (**c**) Western blot showing the effect of the various mutants on T7-GFP or CMV-GFP expression. HEK293-T7 cells were co-transfected with wild type viperin or the viperin mutants Δ42 N, Δ33 C and S1 and T7-GFP or CMV-GFP. The cells were lysed and analyzed by Western blot with GFP (green) and viperin (red) specific antibodies 24 h post-transfection. Tubulin (red, top) was used as a loading control. The effect of wild type viperin is marked with a white arrow head. Quantification of the bands compared to luciferase control is shown at the top of each panel. Full-length blots are presented in Supplementary Fig. 2.

Viperin has three known functional domains. An amphipathic helix at its N-terminal known to mediate its association to the endoplasmic reticulum (ER) and to lipid droplets^28^. A radical SAM domain containing the Cx_3_Cx_2_C motif important for binding an iron–sulfur cluster responsible for its enzymatic activity^6,7^. The third domain, located at the highly-conserved C-terminal of the protein, was found to mediate interactions with host proteins essential for viperin’s enzymatic activity and antiviral functions^11,18^. To gain insight into the mechanism by which viperin inhibits RNA synthesis, we constructed a series of viperin mutants containing mutations in the above-mentioned functional domains. These mutants contained: Δ42 N a deletion of the N-terminal 42 amino acids (a.a.), Δ33C a deletion of 33 a.a. from the C-terminal and S1, containing a point mutation changing the first cysteine in the Cx_3_Cx_2_C motif into alanine (see scheme in Fig. 2a). Viperin is known to localize to the cytosolic face of the ER^29^ and to lipid droplets^28^. To test the effect of these mutations on the localization of the viperin the mutants were co-trasfected to Huh7 cells with calnexin-GFP as ER marker. Wild type viperin staining showed a typical ER reticular staining, which co-localized with calnexin and dot like structures that are most likely lipid droplets (Fig. 2b and Supplementary Fig. S2a). Viperin S1 and ∆33C mutants showed a generally similar localization pattern, although ∆33C was also partly localized to mitochondria (Supplementary Fig. S2b). In contrast, ∆42 N showed a more diffused cytoplasmic and nuclear staining. This result is in agreement with previous studies recognizing this amphipathic helix as the domain that mediates its ER localization^29^. To test for the ability of these mutants to affect T7-GFP expression, we co-transfected these mutants into HEK293-T7 cells together with T7-GFP or CMV-GFP plasmids. Interestingly, only wild type viperin, but not the other mutants, affected T7-GFP expression (Fig. 2c), indicating that all these functional domains are important for viperin’s effect on RNA synthesis. Importantly, none of these mutations influenced the levels of GFP expressed from a CMV promoter.

### Viperin inhibition of T7-mediated expression is not at the translational level

**Figure 3:**
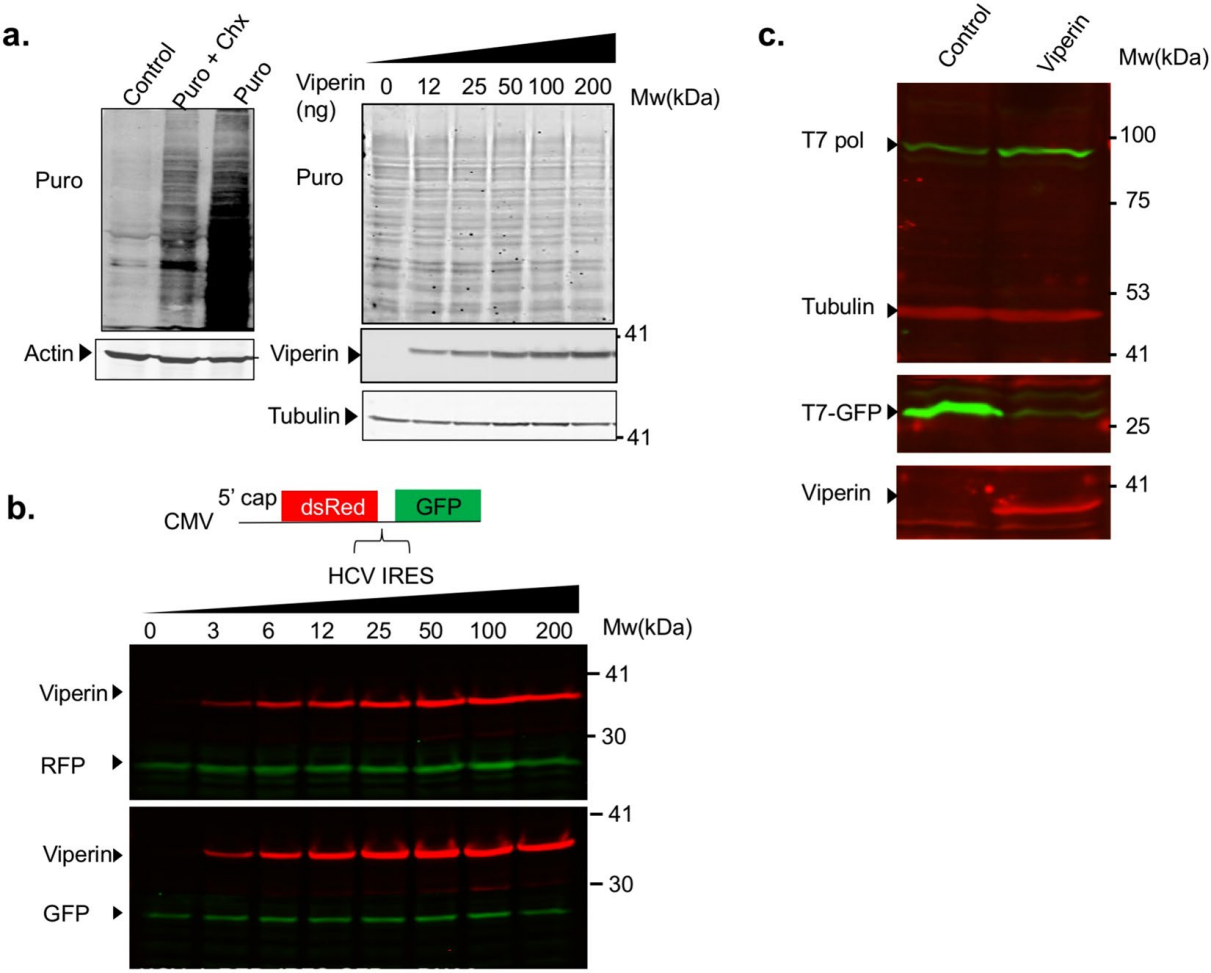
The inhibition of T7-GFP expression by viperin is not at the translational level. (**a**) Viperin does not affect global protein synthesis. HEK293T cells were seeded in a 24-well plate. The next day the cells were transfected the indicated amounts of a viperin expressing plasmid and incubated for 48 h. The cells were then incubated at 37 °C in the presence of puromycin followed Western blotting with puromycin, viperin and tubulin specific antibodies. Cycloheximide (Chx) was used as a negative control. (**b**) Viperin does not inhibit cap or IRES dependent translation. HEK293-T7 cells were co-transfected with the indicated increasing amounts of viperin and a bicistronic plasmid expressing dsRed in a cap dependent manner and GFP from an IRES. The cells were lysed and analyzed by Western blot with viperin (red), GFP or RFP antibodies (green) 24 h post-transfection. (**c**) Viperin does not affect T7-polymerase protein levels. BHK-21 cells were seeded to 24-wells plate. The next day the cells were co-transfected with viperin or empty plasmid (50 ng/well) together with T7 polymerase and T7-GFP (400 ng/well). The cells we’re lysed and analyzed by Western blot 48 h post-transfection using T7 polymerase (green), tubulin (red), GFP (green) and viperin (red) antibodies. Full-length blots are presented in Supplementary Fig. 3.

The reduction in T7-GFP might occur on the translational level, a strategy known to be used by other ISGs^30^. To test this possibility, global protein synthesis rate in the presence of viperin was tested using puromycin labeling^31^. Increasing amounts of viperin were transfected into HEK293T cells. After transfection (48 h), the cells were incubated with puromycin, after which they were washed, lysed and analyzed by Western blot using puromycin-specific antibodies. Fig. 3a (left panel) shows a control experiment with unlabeled, puromycin-labeled and cyclohexamide-treated puromycin-labeled cells. The right panel shows the viperin-transfected cells. A 1-hour chase step was added in this experiment to enable clearer visualization of puromycin staining. Viperin did not affect puromycin labeling pattern, indicating that global protein synthesis rate is not affected. Transcripts expressed from the T7-GFP vector, are translated from an internal ribosome entry site (IRES), while in the CMV-GFP expressing vector, GFP translation begins from a 5′ cap. Thus, the difference in the effect of viperin on the expression of GFP from these two plasmids could be mediated by inhibition of IRES dependent translation. To test this possibility, we used a bicistronic vector expressing dsRed under the control of a CMV promoter, translated from a 5′ cap, followed by the coding region for GFP preceded by the HCV IRES^32^ (Fig. 3b). The vector was transfected into cells that were transfected with increasing amounts of viperin 24 h before. Viperin did not affect dsRed or GFP expression, indicating that neither cap nor IRES dependent translation are inhibited by viperin. One concern is that viperin affects T7-polymerase protein levels. To test this possibility, we co-transfected an empty plasmid or viperin into BHK-21 cells together with T7 polymerase and T7-GFP. Protein levels in cell lysates were analyzed by Western blot 48 h post transfection with specific antibodies (Fig. 3c). While a clear inhibition of T7-GFP could be observed, viperin did not reduce T7 polymerase protein levels. These results demonstrate that the inhibition of T7-GFP expression is not at the translation level.

### Inhibition by viperin is not affected by T7-polymerase localization

**Figure 4:**
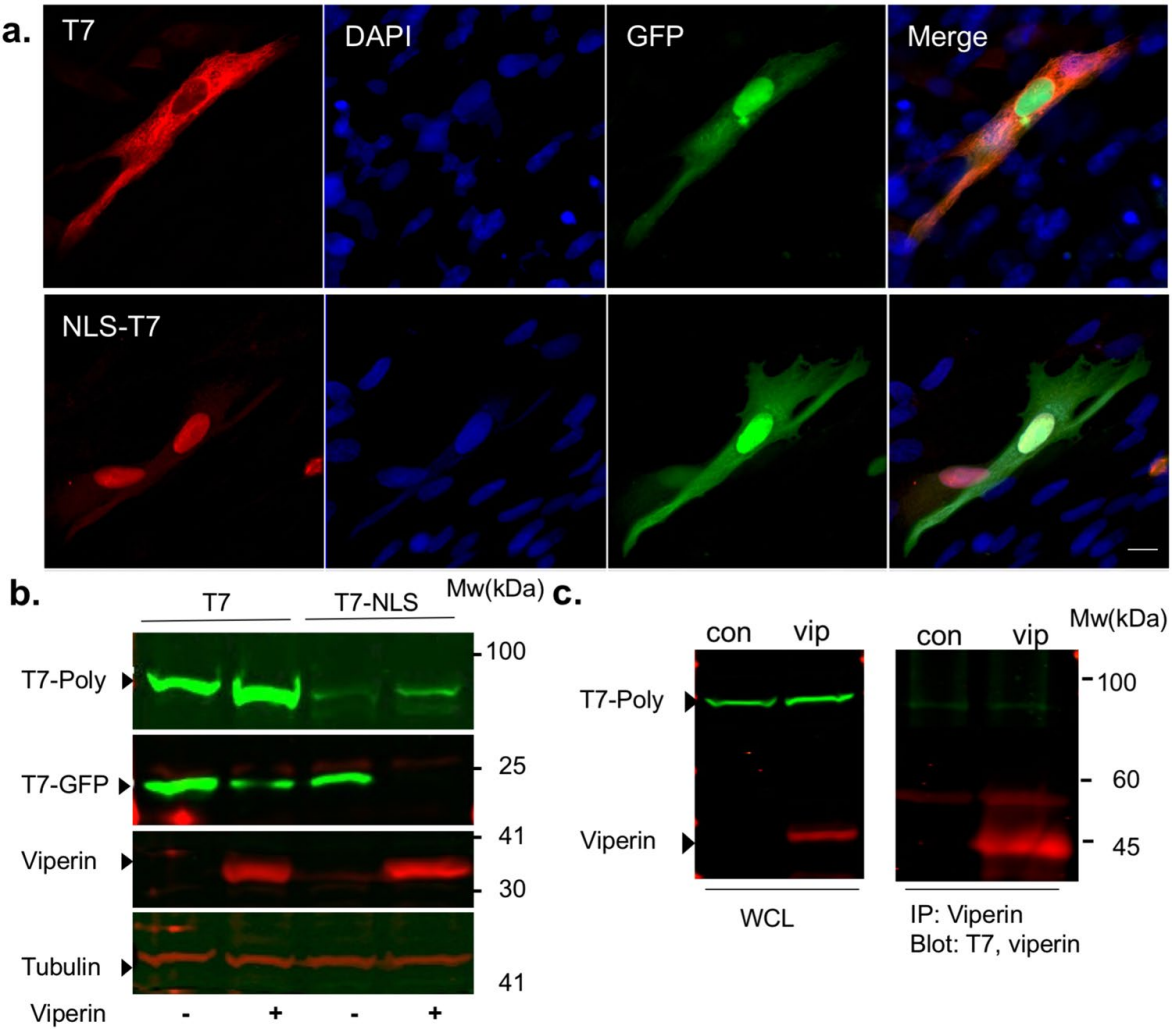
Inhibition by viperin is not affected by T7-polymerase localization. (**a**) Intracellular localization of the NLS-T7-polymerase. Fluorescent microscopy images of BHK-21 cells co-transfected with T7-GFP and either T7-polymerase or NLS-T7-polymerase. Images were obtained 48 h post-transfection Bar = 10 µM. (**b**) The effect of viperin on NLS-T7 polymerase. BHK-21 cells were co-transfected with viperin, T7-GFP and either T7 polymerase or NLS-T7-polymerase. The cells were analyzed by Western blot using the indicated specific antibodies 48 h post transfection. (**c**) HEK293-T7 cells were transfected with viperin or a control plasmid as indicated. Cell lysates were subjected to immunoprecipitation 48 hours later, using A/G agarose beads and viperin specific antibody. Western blot analysis was then performed using anti-Viperin and anti-T7 antibodies. Full-length blots are presented in Supplementary Fig. 4.

Our results suggest that viperin inhibits transcription from the cytoplasmic localized T7-polymerase while CMV-GFP expression mediated by the nuclear RNA polymerase II is not affected at the viperin concentration used. In addition, viperin had no effect when *in vitro* transcribed viral RNA was transfected into viperin expressing cells (not shown), suggesting that viperin does not affect pre-synthesized RNA. A nuclear localized T7-polymerase was generated to examine if there is a direct interaction between viperin and T7 polymerase. In addition this construct was used to test if viperin-mediated inhibition is limited to a specific localization of the T7-polymerase. The nuclear T7-polymerase was generated by introduction of a nuclear localization signal (NLS) into the N-terminal of the T7-polymerase. The NLS-T7-polymerase expressing plasmid was transfected into BHK-21 cells together with T7-GFP and its intracellular localization was determined using a T7-polymerase specific antibody (Fig. 4a). While the wild type T7-polymerase is mostly cytoplasmic, a small number of cells with nuclear staining could be observed, despite its relatively large size (100 kDa). Previous studies have shown that proteins around this size are able to diffuse through the nuclear pore complex^33^. In contrast, the NLS- T7-polymerase was mostly localized to the nucleus, although a weak cytoplasmic staining could also be observed. The NLS-T7-polymerase remained functional as indicated by the T7-GFP fluorescence (Fig. 4a). To test the effect of viperin on the NLS-T7-polymerase, viperin or an empty plasmid were transfected into BHK-21 cells together with either T7 or NLS-T7 polymerase and T7-GFP. The cells were analyzed by Western blot 48 h post-transfection using specific antibodies (Fig. 4b). Viperin inhibited expression from both polymerases regardless of their localization. Immunoprecipitation was performed to test if viperin directly interacts with the T7 polymerase (Fig. 4c). Immunoprecipitation of viperin with a specific antibody did not result in pull down of T7 polymerase. These results indicate that these two proteins probably do not interact directly.

### Viperin affects cytoplasmic RNA levels

**Figure 5:**
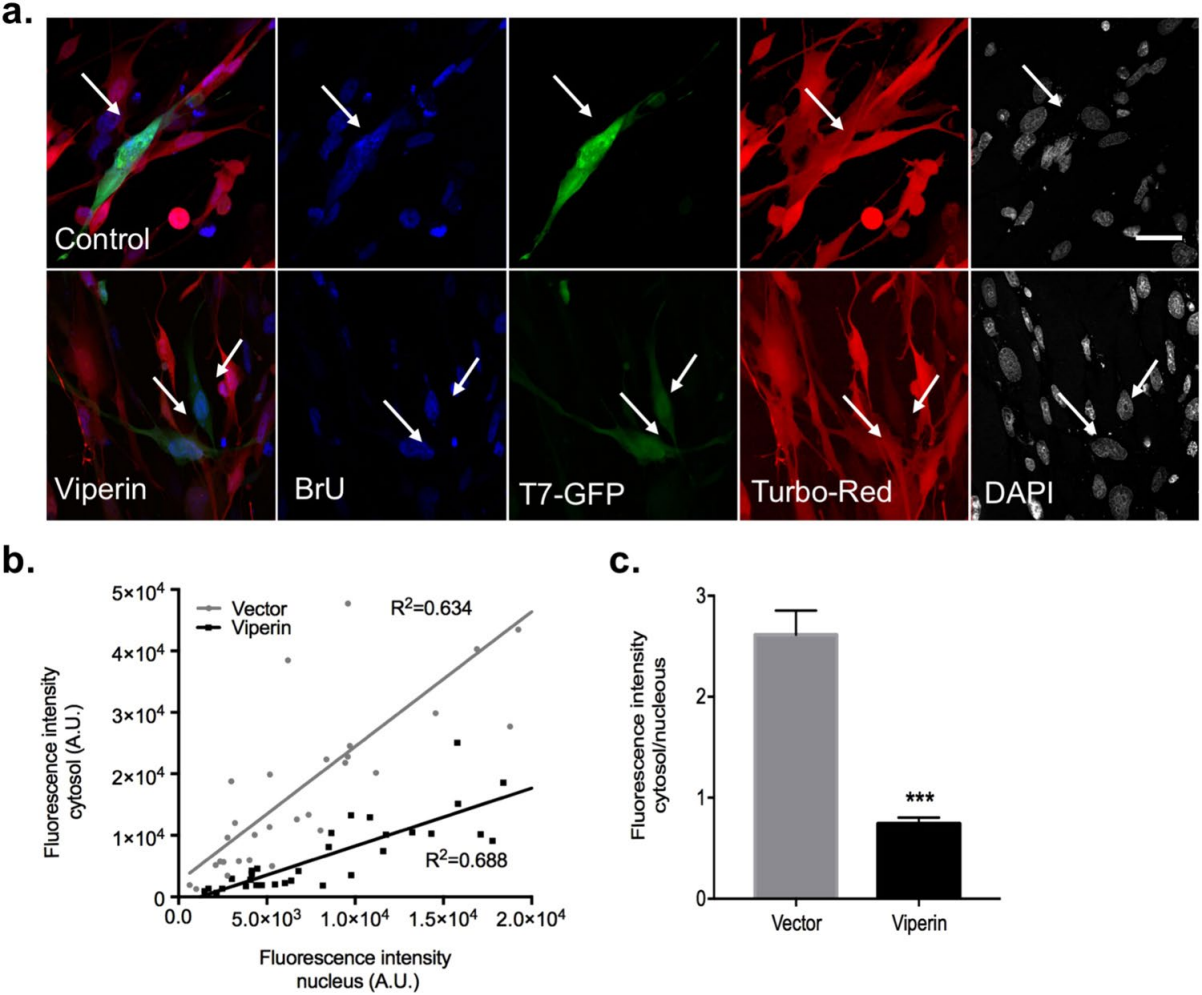
Viperin inhibits T7 polymerase mediated RNA synthesis. (**a**) BSR-T7 cells were infected with bicistronic lentiviruses co-expressing either viperin or luciferase and RFP as a transfection control. The cells were transfected with T7-GFP 48 h post-infection and with BrU after additional 24 h. (**b**) A graph presenting cytosolic vs nuclear fluorescence intensity in vector transfected or viperin transfected individual cells. (**c**) Quantification of the results presented in B. Results are mean ± standard error of the cytoplasm to nuclear fluorescence intensity ratio from 31 cells in each treatment (***p < 0.0001, Student t-test). Bars = 20 µM.

Based on the aforementioned results and on published data regarding viperin’s role in inhibition of RNA virus replication^11,13,17,18,34,35^, we hypothesized that viperin might affect cytoplasmic RNA levels. To test the effect of viperin on RNA synthesis, 5′-bromouridine 5′-triphosphate (BrUTP) incorporation into newly synthesized RNA experiments were performed. This method enabled us to visually distinguish between RNA synthesis in the nucleus and the cytoplasm. To establish the system, control or high multiplicity of infection (MOI) Vesicular stomatitis virus (VSV) infected BHK-21 cells were used. Following infection, the cells were transfected with BrUTP, incubated at 37 °C and then fixed. BrUTP containing RNA was detected using a bromouridine specific antibody. Actinomycin D (ActD), an inhibitor of DNA dependent RNA transcription, was used as a negative control^36^. As expected, control uninfected cells displayed only nuclear staining that was abolished by the addition of ActD (Supplementary Fig. S5). In contrast, both nuclear and cytoplasmic staining were observed in VSV infected cells while only cytoplasmic staining was observed in VSV infected cells treated with ActD. These results confirm the validity of this system. To test the effect of viperin on RNA synthesis, we infected BSR-T7 cells with lentiviral vectors expressing either viperin or luciferase as a control and RFP, followed by BrUTP incorporation experiments. Representative cells from such an experiment are presented in Fig. 5b. In control cells, both cytoplasmic and nuclear staining was observed. In contrast, in viperin expressing cells, mainly nuclear staining could be detected (Fig. 5a). Fluorescent intensity of the nuclear and cytoplasmic staining was quantified in viperin-expressing (n = 31) and control cells (n = 31) from each treatment (Fig. 5b,c). Cytoplasmic staining was significantly lower in viperin expressing cells compared to controls. This can be clearly observed from the significantly lower cytoplasm to nuclear fluorescence intensity ratio (p < 0.001, student’s T-Test). These results indicate that there are lower cytoplasmic RNA levels in these cells, suggesting that viperin inhibits RNA synthesis mediated by the cytoplasmic T7 polymerase.

## Discussion

Viperin is a potent broad-spectrum antiviral protein with a yet unknown mechanism. Viperin was found to inhibit expression from T7-polymerase while other ISGs did not specifically affect T7-GFP expression. Viperin did not inhibit cap or IRES dependent translation, nor did it affect T7 polymerase protein levels, indicating that its effect might be on transcription. BrUTP incorporation experiments confirmed this hypothesis showing significantly lower BrUTP levels in the cytoplasm of cells expressing viperin compared to controls, suggesting that viperin affects RNA stability or synthesis. Of note, higher levels of viperin expression do show some effects on nuclear RNA synthesis as well. It is still not clear what is the mechanistic basis for the larger effect observed at the cytoplasm. One possibility is that under the conditions used, most of the cellular transcription activity that occurs is the robust T7-GFP expression or cytoplasmic viral replication and thus, this is the activity that is more affected. Another possibility is that the observed effect of viperin is mediated by degradation of the newly produced cytoplasmic RNA following its synthesis. Transfection of viral RNA into viperin-expressing cells did not affect its further translation (not shown). Furthermore, viperin-expressing cells show lower levels of BrUTP expression 1 h post BrUTP transfection. An effect within such a time frame is most likely on RNA synthesis rather than degradation.

The question of the relevance of viperin’s activity against the T7-dependent expression to mammalian biology should be raised. The T7 polymerase system is a widely-used system to initiate cytoplasmic replication in reverse genetics systems of many viruses^37–42^. Thus, T7 polymerase is fully functional in mammalian cells and in can generate high levels of transcription in the cytoplasm of many cell types confirming its relevance. Additional support for its relevance comes from the finding that viperins are highly conserved and organisms from fungi to bacteria and archaebacteria also express viperin-like enzymes^21^. Moreover, human viperin was found to cause radical SAM- dependent elongation of *E. Coli* indicating that its substrate might be shared between bacteria and humans^43^.

While viperin was previously shown to affect RNA replication of several viruses using real-time PCR, it is still unclear how this occurs^11,13–18^. Viperin was shown to bind to viral non-structural proteins associated with viral replication^13,14,17^. The observed inhibition of the T7 polymerase might indicate a different mechanism since in this system RNA synthesis does not occur in membrane complexes or requires additional bacteriophage proteins. BrUTP experiments in viral systems were so far unsuccessful due to low efficiency of transfection in the infected cells. Nevertheless, this highlights the significance of our findings in providing a highly tractable system to study this activity of viperin. If viperin indeed inhibits RNA synthesis, it seems to use a mechanism that inhibits a rather broad range of polymerases; RNA-dependent RNA polymerases from different viruses and a bacteriophage DNA-dependent RNA polymerase. Thus, it is less likely that viperin directly binds these structurally different RNA polymerases. In addition, our immunoprecipitation experiments, which do not detect such an interaction, suggest otherwise. An *in vitro* assay would be useful in the identification of a direct effect of viperin on the polymerase. However, since viperin is a membrane-associated protein^28^, requires an additional protein partner for its activity^11^, might require addition of nucleoside precursors^21^ and needs to be purified under anaerobic conditions, establishment of such an *in vitro* assay will be extremely challenging. One possible option is that viperin inhibits a host cell factor essential for viral/cytoplasmic RNA synthesis (for examples of such factors see^44,45^). Alternatively, viperin might affect a general RNA synthesis resource that is more frequently utilized during high levels of RNA synthesis. For example, viperin might affect nucleoside synthesis or modify a nucleoside to create an antiviral nucleoside analogue. Support for the latter possibility may come from a recent finding that viperin’s substrate is a nucleoside triphosphate^21^. In addition, Oxetanocin A, a nucleoside analogue produced in bacteria with an antiviral activity is produced by a SAM radical family member^20^. Alternatively, a subset SAM radical enzymes were found to catalyze methylation reactions^46^. For example, RlmN and Cfr post-transcriptionally methylate ribosomal RNA nucleotides^47^. Since neither cap nor IRES-dependent translation is inhibited by viperin (Fig. 3), another possibility is that viperin might induce some RNA modification affecting RNA stability. Experiments attempting to rescue RNA synthesis in viperin-transfected cells using purines or pyrimidines both in the T7 system and with different viperin-inhibited RNA viruses yielded inconclusive results. The recently published structure of mouse viperin suggests that the substrate of viperin may be a nucleoside triphosphate^21^. It was also suggested that the active site better reflects a binding site for a free or chain-terminal nucleotide than a nucleotide in a nucleic acid chain. According to this hypothesis a chain terminal nucleotide might be a 5′-cap, our findings, however do not support this possibility as T7-GFP is not translated from a cap structure. Thus, our current results suggest that most favorable hypothesis is that viperin produces a nucleoside analogue with antiviral activity like Oxetanocin A^20^.

## Methods

### Cell culture viruses and transfections

Vero, HEK293T, HEK293-T7, BSR-T7 cells were propagated in Dulbecco’s Modified Eagle’s Medium (DMEM) supplemented with 10% (v/v) fetal calf serum, 1% (v/v) penicillin/streptomycin (Biological Industries, Bet-Haemek, Israel). The HEK293-T7 and BSR-T7 cells were grown in the presence of hygromycin (Invivogen, San Diego, CA, 0.2 g/ml) or G418 (Calbiochem, 0.7 g/ml) respectively. Lipofectamine 2000 (Invitrogen, Carlsbad, CA) or polyethyleneimine (Polysciences, Warrington, PA) were used for plasmid DNA transfections of subconfluent cells. Vesicular stomatitis virus (VSV) strain ts045 was propagated and tittered in Vero cells as described^48^.

### Plasmids

T7-GFP and the T7 polymerase expressing plasmids were a kind gift from Prof. Shmuel Rozenblatt (Tel-Aviv University). The c-Myc NLS signal was added to the N-terminus of the T7 polymerase by PCR using the following primers: FW: CCAAGCGAGTTAAATTAGACAATACCATAAACATTGCTAAGAACG. RV: CAGCAGGCATGGGTACCCGGTGTCTTCTATGGAGGTC. The original plasmid was eliminated using the DpnI enzyme, the PCR product containing the entire original plasmid plus the NLS signal was gel-purified, ligated and transformed into bacteria. pEGFP-C1 (Clontech, Palo Alto, CA), was used to express GFP from a CMV promoter. The T7-firefly luciferase and the ß-galactosidase expressing plasmids were from Promega (Madison, WI). HEK293-T7 a pool of T7 polymerase expressing HEK293 cells was a gift from Prof. Eran Bacharach (Tel-Aviv University). Calnexin-GFP was a gift from Prof. Rina Arbesfeld (Tel-Aviv University). For the T7-GFP and T7-luciferase quantification experiments human viperin was cloned into pCAGGS plasmid using Gibson assembly. Briefly, viperin was amplified from a viperin-F.LAG plasmid (a kind gift from Prof. Yi-Ling Lin, Academia Sinica, Taiwan) with the primers primers Fw 5′-GAATTCGAGCTCATCGATGCATGGTACCATGTGGGTGCTTACACCTGCTG-3′ for Fw and Rev 5′-CATAATTTTTGGCAGAGGGAAAAAGATCTCTACCAATCCAGCTTCAGAT C-3′ and ligated into pCAGGS digested with KpnI and BglII. The viperin mutants were similarlly cloned into pCAGGS. The primers used for Δ42 N were Fw 5′-GAATTCGAGCTCATCGATGCATGGTACCATGCTAGCTACCAAGAGGAG-3′. For Δ33 C Rev 5′-ATAATTTTTGGCAGAGGGAAAAAGATCTCTATTCATCCAGAATAAGGTAG-3′. For the S1 mutant two overlapping mutation-containing viperin fragments used for Gibson assembly into the afore-mentioned sites. The primer used for the first fragment: Fw 5′-GAATTCGAGCTCATCGATGCATGGTACCATGTGGGTGCTTACACCTGCTG-3′, Rev 5′-GCGAAGCCGGCTTTGTAGTTGGCCTGGCGAGTGAAGTGATAGTTG-3′ and for fragment 2: Fw 5′-GCCAACTACAAAGCCGGCTTCGCTTTCCACACAGCCAAAACATC-3′ Rev 5′-CATAATTTTTGGCAGAGGGAAAAAGATCTCTACCAATCCAGCTTCAGATC-3′. The CFP-YFP fusion expression plasmid was a gift from Prof. Koret Hirschberg (Tel-Aviv University). ISG expressing lentiviruses *pTRIP*.*CMV*. *IVSb*.*ISG*.*ires*.*TagRFP* were a gift from Prof. Charlie Rice^25^ (Rockefeller university). The bicistronic plasmid expressing dsRed in a cap dependent manner and GFP from an IRES^32^ was a kind gift from Prof. C.G Proud (University of Dundee).

### Western blot analysis

Cell were lysed in lysis buffer containing 300 mM NaCl, 100 mM Tris–HCl pH 8, 0.2 mM EDTA, 10% Glycerol, supplemented with a protease inhibitor cocktail (Sigma, Israel). The lysates were separated using sodium dodecyl sulfate-polyacrylamide gel electrophoresis (SDS-PAGE) and transferred to a nitrocellulose membrane. Membranes were blocked for 1 h in blocking solution containing 5% non-fat milk (Sigma, Israel) in phosphate buffer saline (PBS) followed by incubation with the appropriate primary antibody. Primary antibodies used were rabbit polyclonal anti-RFP antibody (MBL, Woburn, MA) and a rabbit anti-GFP polyclonal antibody (Abcam, Cambridge, MA). Anti-Viperin Antibody, clone MaP.VIP was from Millipore (Billerica, MA) and anti-T7 polymerase from Creative biolabs, Shirley, NY. Monoclonal mouse antibodies against β actin and tubulin were from Sigma. The blots were incubated with IR-Dye conjugated secondary antibodies (Li-cor, Lincoln, NE) and visualized using a LI-COR infrared imager (Odyssey). Band intensity was quantified using imageJ^49^ and normalized to the loading control. Values are presented as percent of the luciferase control.

### Immunoprecipitation

HEK293-T7 cells were plated to 15-cm plate and transfected the next day with 15 μg of viperin or a control plasmid. The cells were harvested 48 hours post-transfection and lysed with the M2 lysis buffer (50 mM Tris-base (pH 7.4), 50 mM NaCl, 10% glycerol, 1% Triton X-100, 0.5 mM EDTA, 0.5 mM EGTA) supplemented with a protease inhibitor cocktail (Sigma). Cell lysates were incubated for 3 h with anti-viperin antibody conjugated to protein A/G agarose beads (Santa Cruz Biotechnology, Santa Cruz, CA) at 4 °C. Beads were collected by slow centrifugation, washed four times with lysis buffer and analyzed by SDS–polyacrylamide gel electrophoresis followed by detection with the aforementioned antibodies.

### mRNA translation measurements

Puromycin incorporation was used to monitor protein synthesis^31^. Puromycin, is an aminonucleoside antibiotic which is an aminoacyl tRNAs structural analog that is incorporated into the nascent polypeptide chain and prevents its elongation. At low concentrations puromycin incorporation is directly proportionate to the rate of mRNA translation. Immunoblotting with the 12D10 monoclonal antibody to puromycin (Millipore) was used to detect puromycin incorporation. The translation inhibitor cycloheximide (100 μg/ml, Sigma) used as a negative control. Cycloheximide was added to the cells 30 min prior to the puromycin addition. For puromycin labeling the cells were incubated at 37 °C with medium containing 10 µg/ml puromycin for 30 min. The cells were washed with PBS and incubated with fresh media for an additional hour followed by Western blot.

### Immunofluorescence microscopy

Cells were cultured on coverslips, fixed for 30 min with 2% formaldehyde in PBS, permeabilized with 0.2% Triton X-100 in PBS and blocked with PBS + 1%BSA + 0.05% Triton for 1 h. Immunolabeling was performed overnight with the anti-viperin primary antibody (1:200). The secondary antibody was Alexa Fluor 555 goat anti-mouse (1:2000). 4′,6-diamidino-2 –phenylindole (DAPI, Molecular probes, Eugene, OR) was used for nuclei staining. Images were acquired using a Zeiss LSM800 confocal laser-scanning microscope (Carl Zeiss MicroImaging, Jena, Germany) hooked to an inverted microscope.

### *In situ* labeling of newly-synthesized RNA with 5-Bromouridine

5-Bromouridine experiments were performed as described^50^. Briefly BSR-T7 cells grown on cover slips were left untreated or infected with VSV at the MOI of 3. For viperin experiments the cells were infected with a viperin and RFP expressing lentivirus and transfected with T7-GFP or vector control 24 h later. Following infection or transfection (24 h) the cells were depleted of uridine, by a 1 h incubation in low glucose DMEM (Invitrogen) supplemented with 20 mM D-glucosamine (Sigma). The cells were then transfected with 0.5 mM BrUTP (Sigma) incubated at 37 °C for 1 hour to allow the BrUTP incorporation. After the incubation, the cells were washed with PBS, fixed with 2% PFA, followed by 3 min ice-cold methanol, permeabilized and stained as previously described. RNA was detected using an antibody against bromodeoxyuridine (mouse monoclonal, clone BMC9318, 1:60). To inhibit cellular transcription cells were treated with 10 µg/ml actinomycin D (ActD, Sigma) 30 min before to BrUTP labeling. The quantitative analysis was performed using the ImageJ. Any cell displaying triple staining of red (viperin or luciferase control), GFP and blue (brU) was analyzed. The fluorescence intensity (FI) was calculated by multiplying the area of region of interest (i.e total cell or nucleus) by the average fluorescence intensity of this region. The cytosolic fluorescence intensity was calculated by subtraction of nuclear FI from total cell FI.

### Data availability

No datasets were generated or analyzed during the current study.

## Electronic supplementary material

Supplementary Information
