# Supplementary material for: The antiviral protein Viperin suppresses T7 promoter dependent RNA synthesis–possible implications for its antiviral activity
Source: Sci Rep. 2018 May 25;8:8100. doi: 10.1038/s41598-018-26516-z (PMC5970183; doi:10.1038/s41598-018-26516-z)
Supplement: Supplementary file 1 — Supplementary Information [file 41598_2018_26516_MOESM1_ESM.pdf]

The antiviral protein Viperin suppresses T7 promoter dependent RNA synthesis–possible implications for its antiviral activity

Anna Dukhovny[1], Amir Shlomai[2], Ella H. Sklan[1*].

Supplementary material

a.

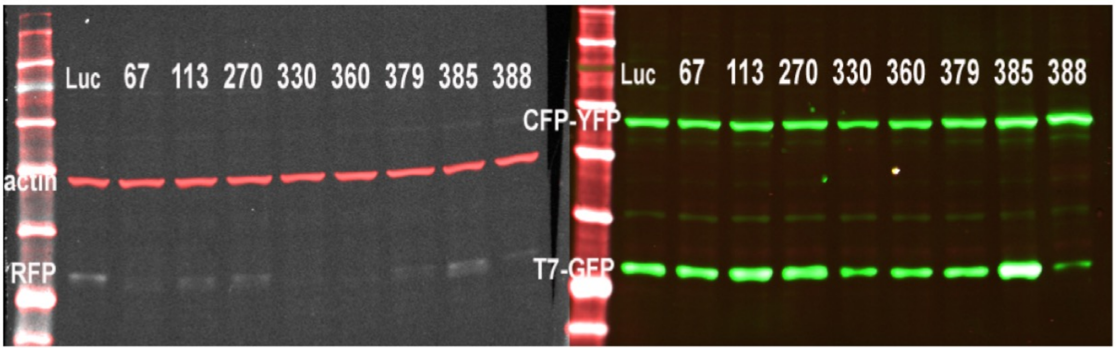

b.

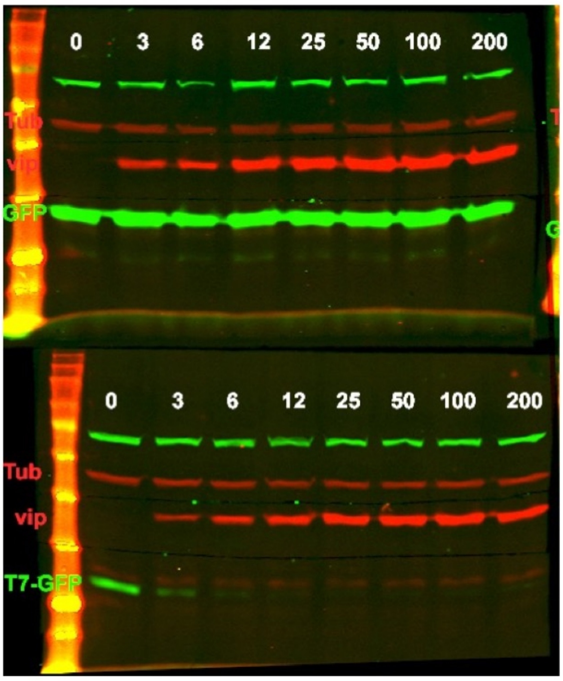

e.

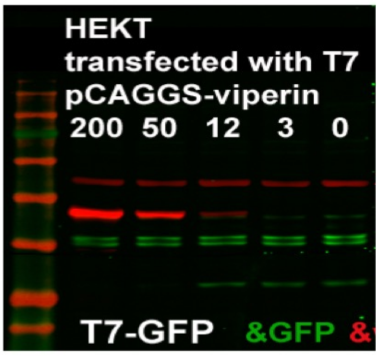

Supplementary figure 1: Uncropped odyssey Li-cor files for the blots presented in figure 1a,b and e.

a.
b.
c.
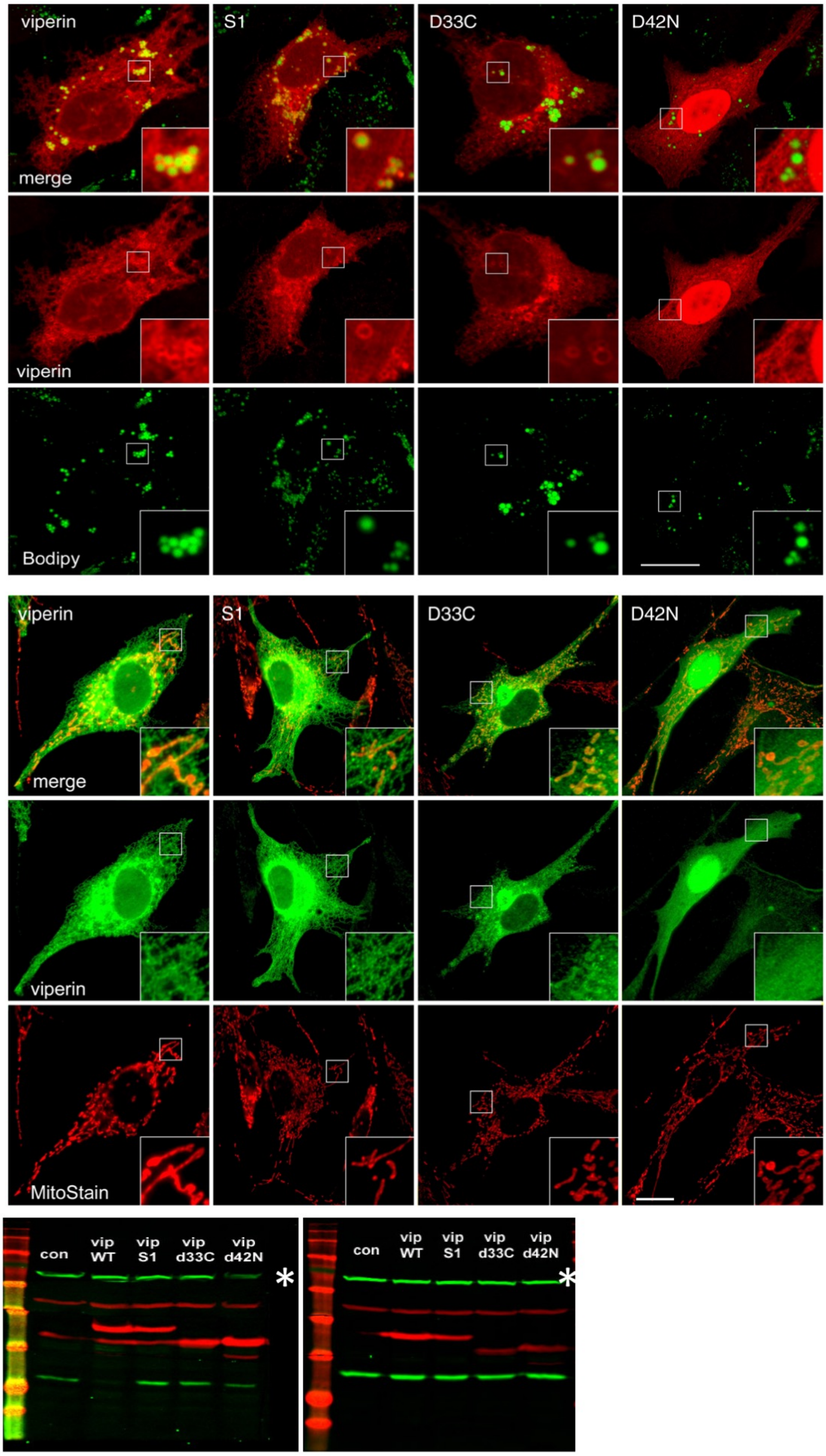

Supplementary figure 2: a,b Subcellular Localization of the viperin mutants. Viperin mutants were transfected into Huh7 cells 24 h post transfection the cells were stained with Mitostain (a, BioRad) or Bodipy 493/503 (b, Molecular probes). The cells were fixed and immunostained using a viperin antibody. Bar= 20μm **c.** Uncropped odyssey Li-cor files for the blots presented in figure 2c. The top bands (labeled with an asterisk) are non-specific bands. As can be seen their levels do not change between the samples.

a.

b.

c.

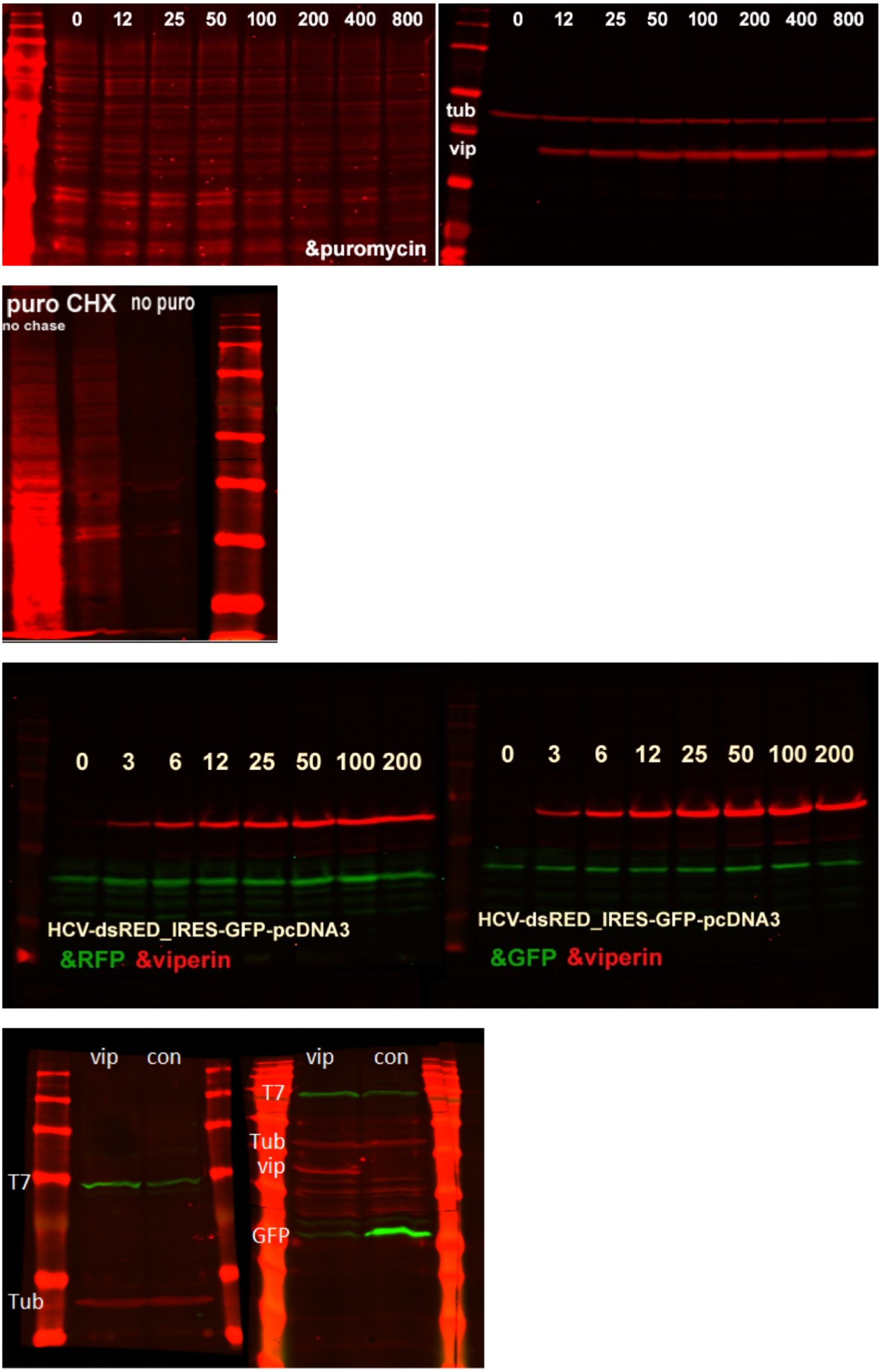

Supplementary figure 3: Uncropped odyssey Li-cor files for the blots presented in figure 3a-c. The blots in a were converted to gray scale in the manuscript to increase clarity.

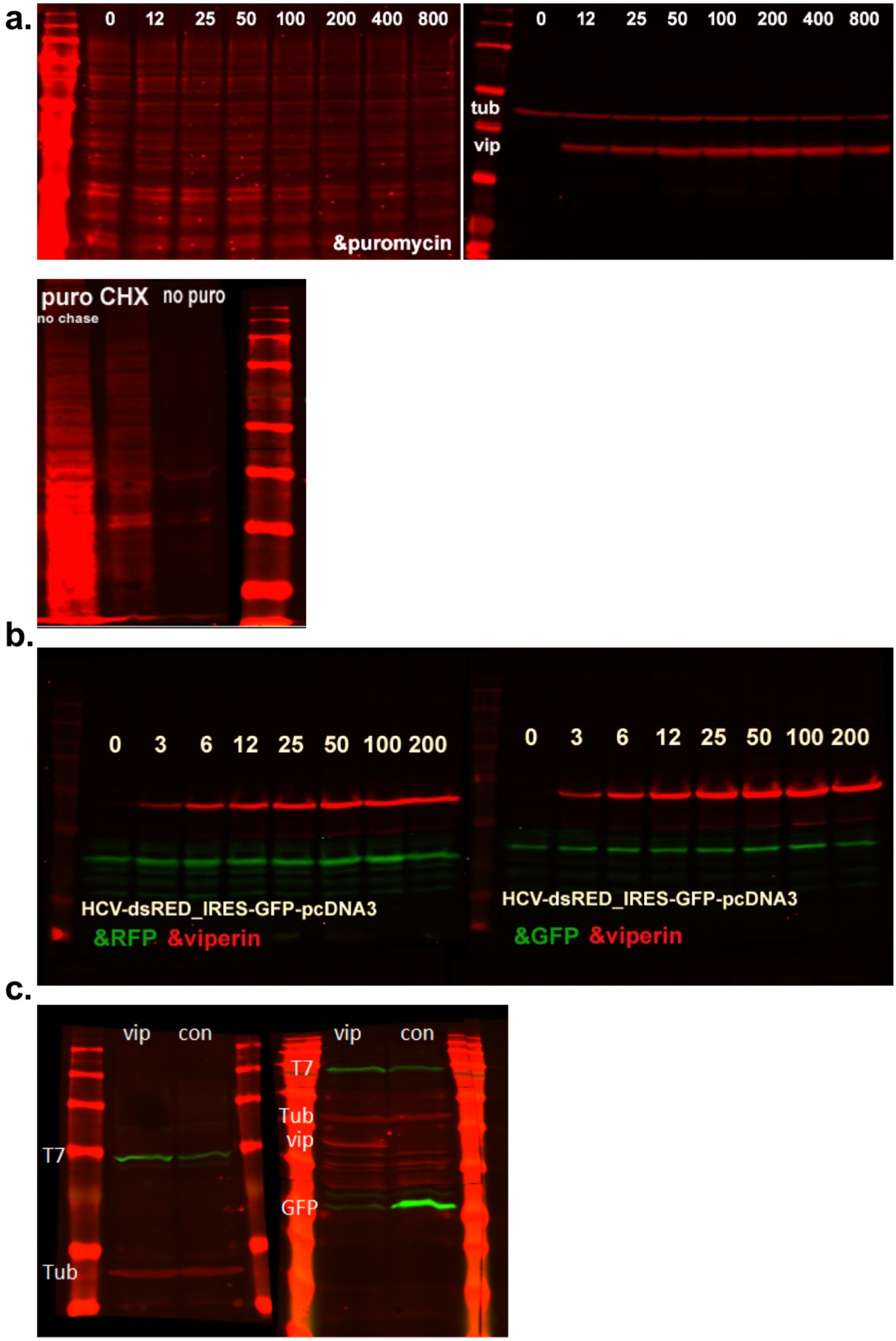

Supplementary figure 3: Uncropped odyssey Li-cor files for the blots presented in figure 3a-c. The blots in a were converted to gray scale in the manuscript to increase clarity.

b.

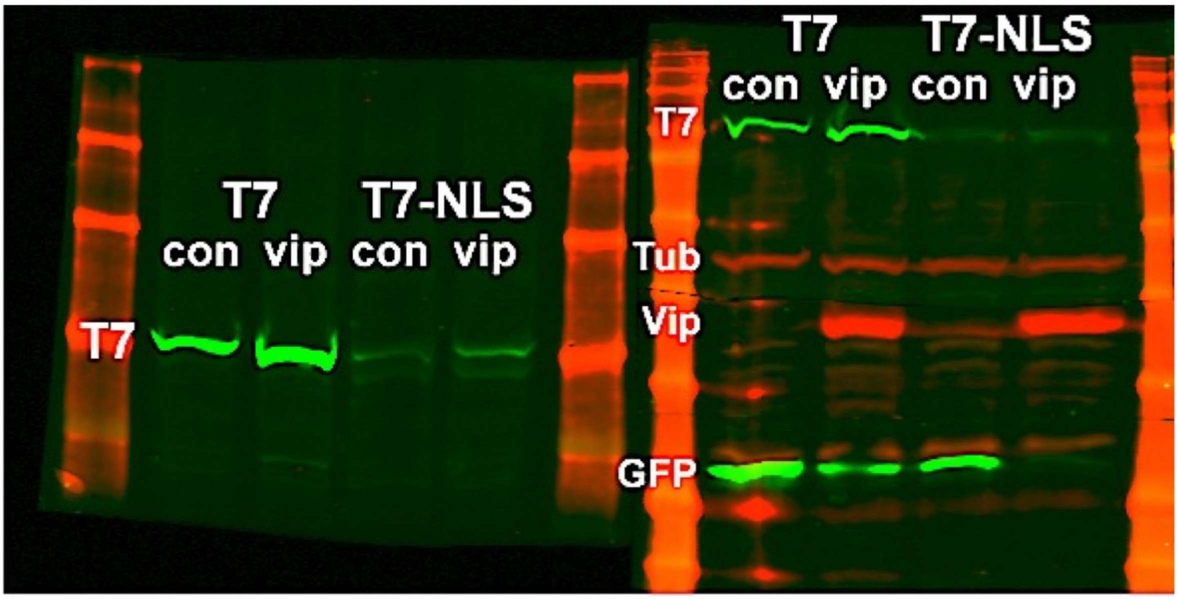

Supplementary figure 4: Uncropped odyssey Li-cor files for the blots presented in figure 4b. The blot on the left side is a rerun of the samples in a lower percentage gel to better visualize the T7-polymerase that has a a higher molecular weight.

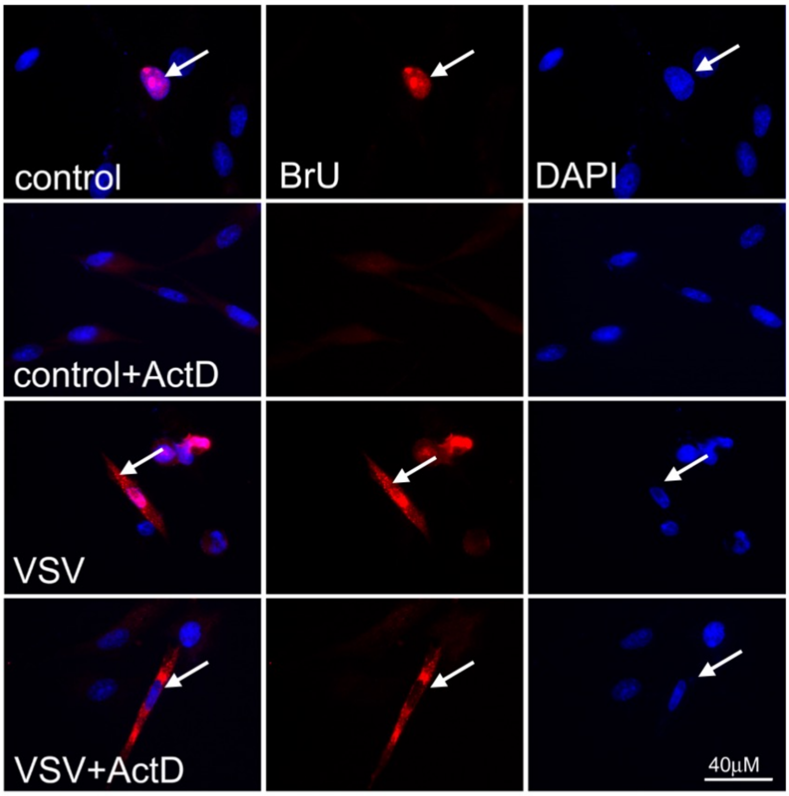

Supplementary Figure 5: Visualization of RNA synthesis in cells. Fluorescent microscopy images of uninfected BSR-T7 cells (Top) showing BrU (red) cell nuclei (DAPI, blue). Actinomycin D a DNA-dependent RNA synthesis inhibitor was used as a control. Lower panels: BSR-T7 cells infected with VSV at an MOI of 3.
